# Supplementary material for: Relative Effectiveness of Cell-Cultured versus Egg-Based Seasonal Influenza Vaccines in Preventing Influenza-Related Outcomes in Subjects 18 Years Old or Older: A Systematic Review and Meta-Analysis
Source: Int J Environ Res Public Health. 2022 Jan 12;19(2):818. doi: 10.3390/ijerph19020818 (PMC8775496; doi:10.3390/ijerph19020818)
Supplement: Supplementary file 1 [file ijerph-19-00818-s001.zip › Figure_S1_v04.pdf]

Figure S1. Other confounders contribution to heterogeneity

Adjusted by confounders	Reported results	aRR with 95% CI	p-value
**Health care contacts**			
No or not reported	4	0.98 [0.91, 1.04]	0.447
Yes	7	0.93 [0.88, 0.98]	0.003
Test of group differences: $Q_b(1) = 1.47$, $p = 0.23$			
**Underlying conditions**			
No or not reported	1	0.95 [0.83, 1.09]	0.475
Yes	10	0.95 [0.91, 0.99]	0.019
Test of group differences: $Q_b(1) = 0.00$, $p = 0.97$			
**Frailty**			
No or not reported	8	0.97 [0.93, 1.02]	0.275
Yes	3	0.91 [0.85, 0.98]	0.007
Test of group differences: $Q_b(1) = 2.41$, $p = 0.12$			
**Previous vaccination**			
No or not reported	7	0.94 [0.90, 0.99]	0.015
Yes	4	0.98 [0.87, 1.09]	0.707
Test of group differences: $Q_b(1) = 0.36$, $p = 0.55$			
**Calendar time**			
No or not reported	9	0.94 [0.90, 0.99]	0.010
Yes	2	1.03 [0.83, 1.28]	0.809
Test of group differences: $Q_b(1) = 0.54$, $p = 0.46$			
**Vaccination date**			
No or not reported	4	1.04 [0.88, 1.22]	0.638
Yes	7	0.94 [0.90, 0.98]	0.008
Test of group differences: $Q_b(1) = 1.32$, $p = 0.25$			
**Statistical method**			
LR	2	0.96 [0.84, 1.10]	0.560
Cox	1	1.06 [0.82, 1.36]	0.660
Not reported	1	0.94 [0.61, 1.45]	0.781
LR+PS	1	1.07 [0.76, 1.52]	0.689
LR+IPTW	2	0.98 [0.89, 1.07]	0.592
IPTW+Poisson	3	0.93 [0.87, 1.00]	0.050
PS+IPTW+Poisson	1	0.89 [0.86, 0.92]	0.000
Test of group differences: $Q_b(6) = 7.56$, $p = 0.27$			

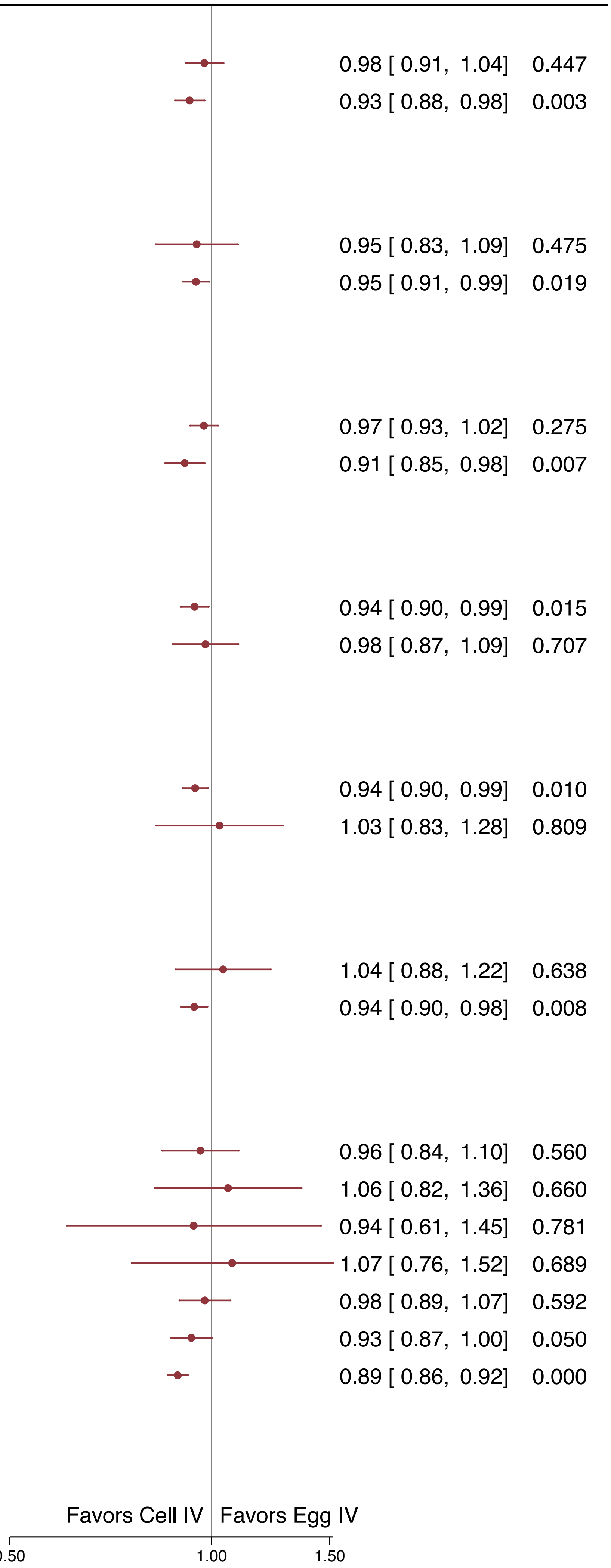

aRR, adjusted relative risk. CI, confidence interval.
Results excluded, motive and identifier:
Outlier: Boikos 2020b. Multiple dependent estimates: Bruxvoort 2019b; Izurieta 2019a; Izurieta 2019c; Klein 2020b; Fu Tseng 2019a; Fu Tseng 2019c; Fu Tseng 2019d; Fu Tseng 2019e; and Izurieta 2020b
Unique estimates in their category: Izurieta 2020c and d (2019-2020 season) and Martin 2020 (age-group ≥ 18).
