# Supplementary material for: Relative Effectiveness of Cell-Cultured versus Egg-Based Seasonal Influenza Vaccines in Preventing Influenza-Related Outcomes in Subjects 18 Years Old or Older: A Systematic Review and Meta-Analysis
Source: Int J Environ Res Public Health. 2022 Jan 12;19(2):818. doi: 10.3390/ijerph19020818 (PMC8775496; doi:10.3390/ijerph19020818)

Figure S3. Graphical and statistical small study bias analysis by season, age group, reported outcome, outcome determination method, study design, funding and risk of bias (I)

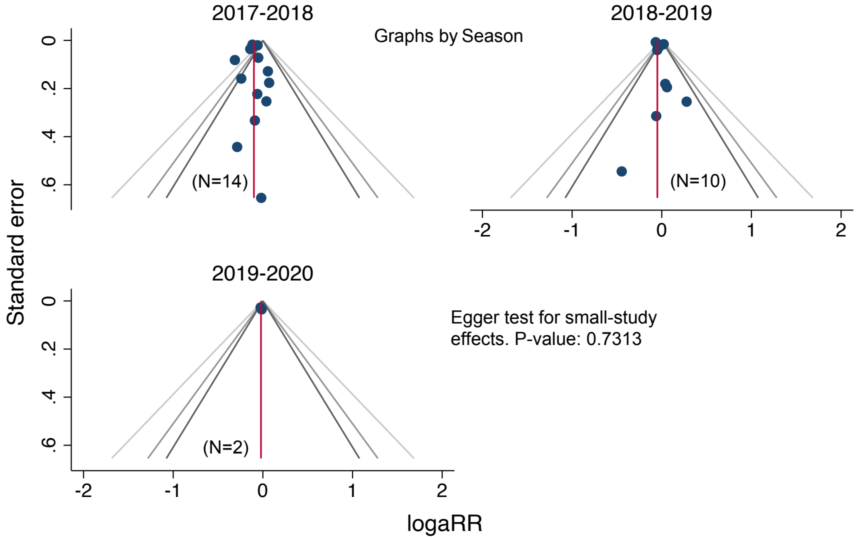

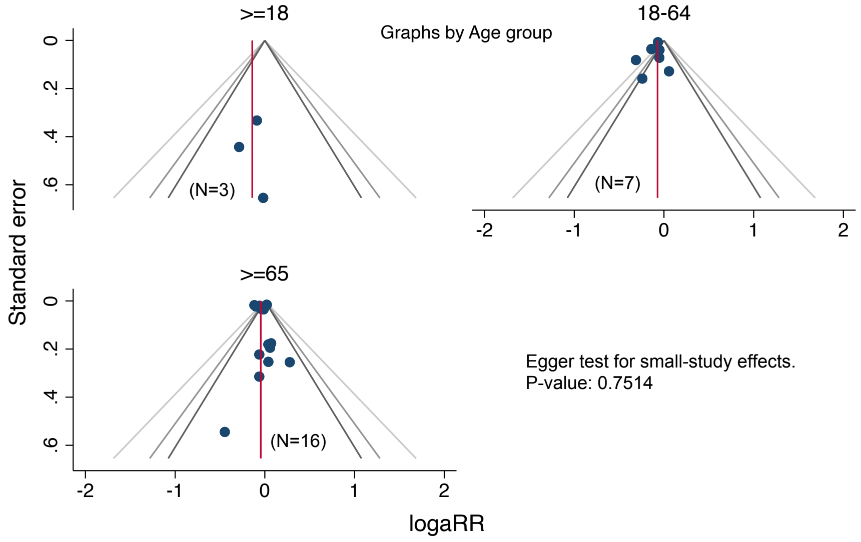

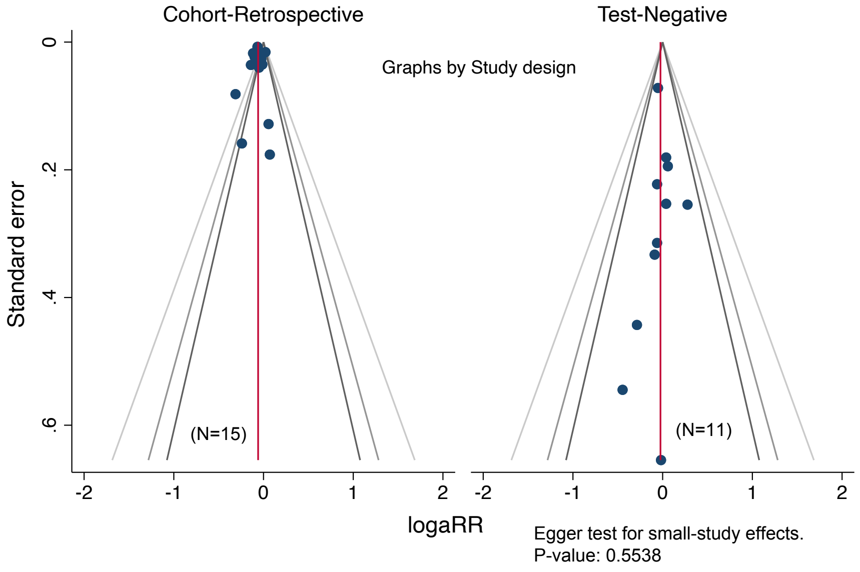

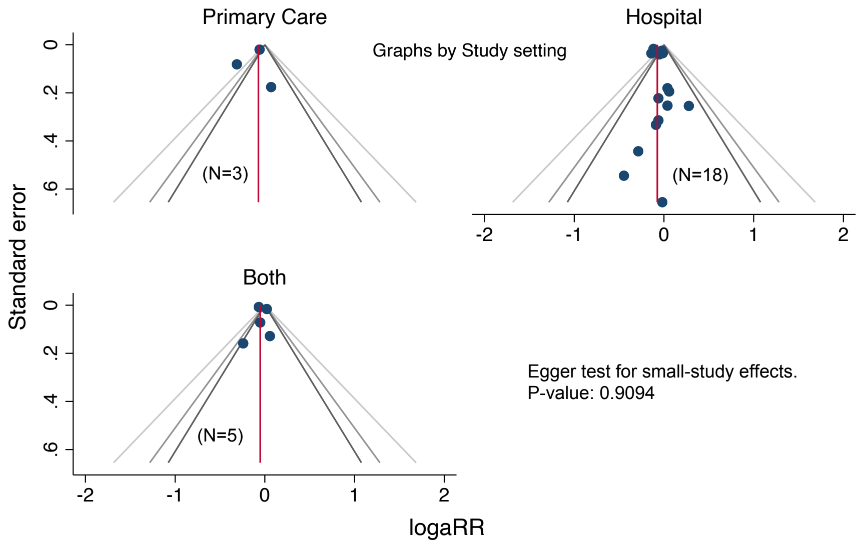

Figure S3. Graphical and statistical small study bias analysis by season, age group, reported outcome, outcome determination method, study design, funding and risk of bias (II)

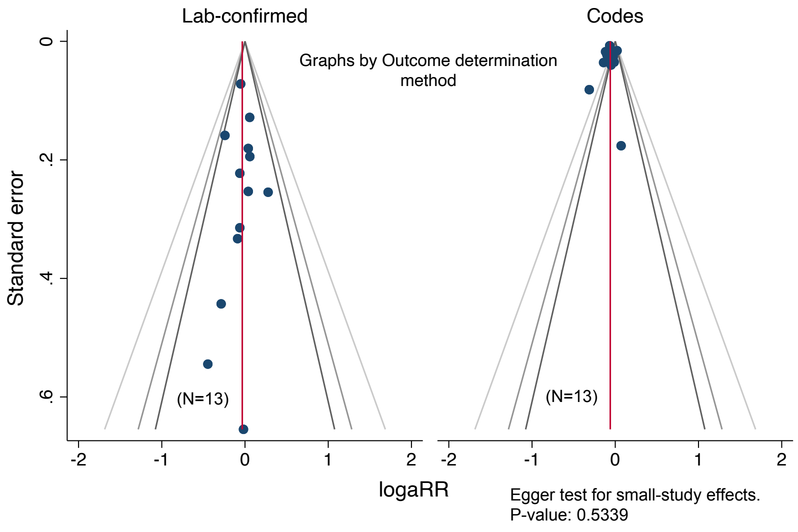

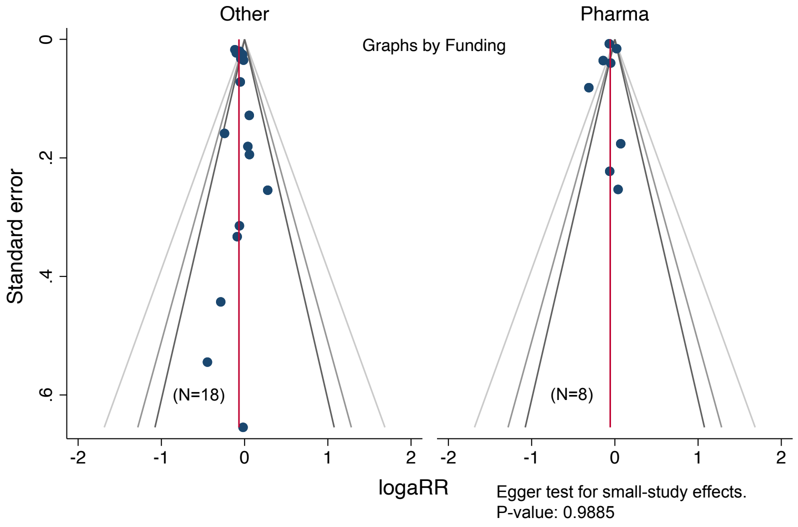

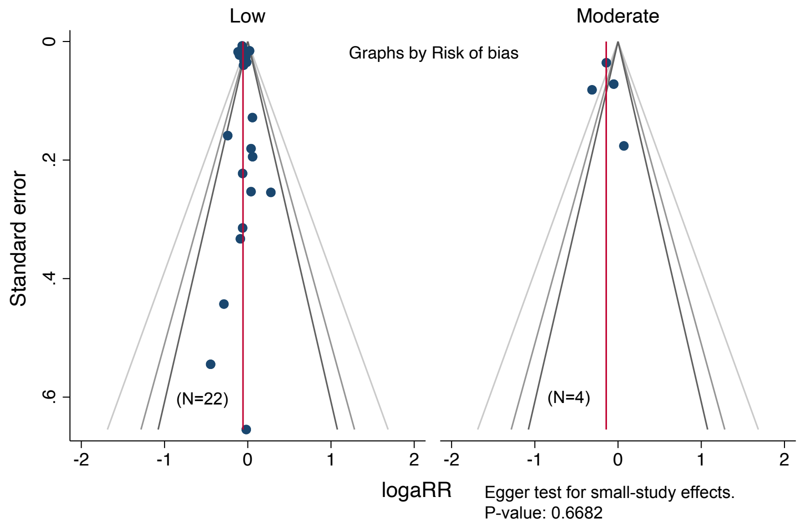

Supplement: Supplementary file 1 [file ijerph-19-00818-s001.zip › Figure_S3 Funnel plots a to g_v04.pdf]
