# Supplementary material for: Relative Effectiveness of Cell-Cultured versus Egg-Based Seasonal Influenza Vaccines in Preventing Influenza-Related Outcomes in Subjects 18 Years Old or Older: A Systematic Review and Meta-Analysis
Source: Int J Environ Res Public Health. 2022 Jan 12;19(2):818. doi: 10.3390/ijerph19020818 (PMC8775496; doi:10.3390/ijerph19020818)

Figure S4. Non parametric trim and fill analysis restricted to the 2017-2018 season.
Multiple dependent and outlier results excluded.

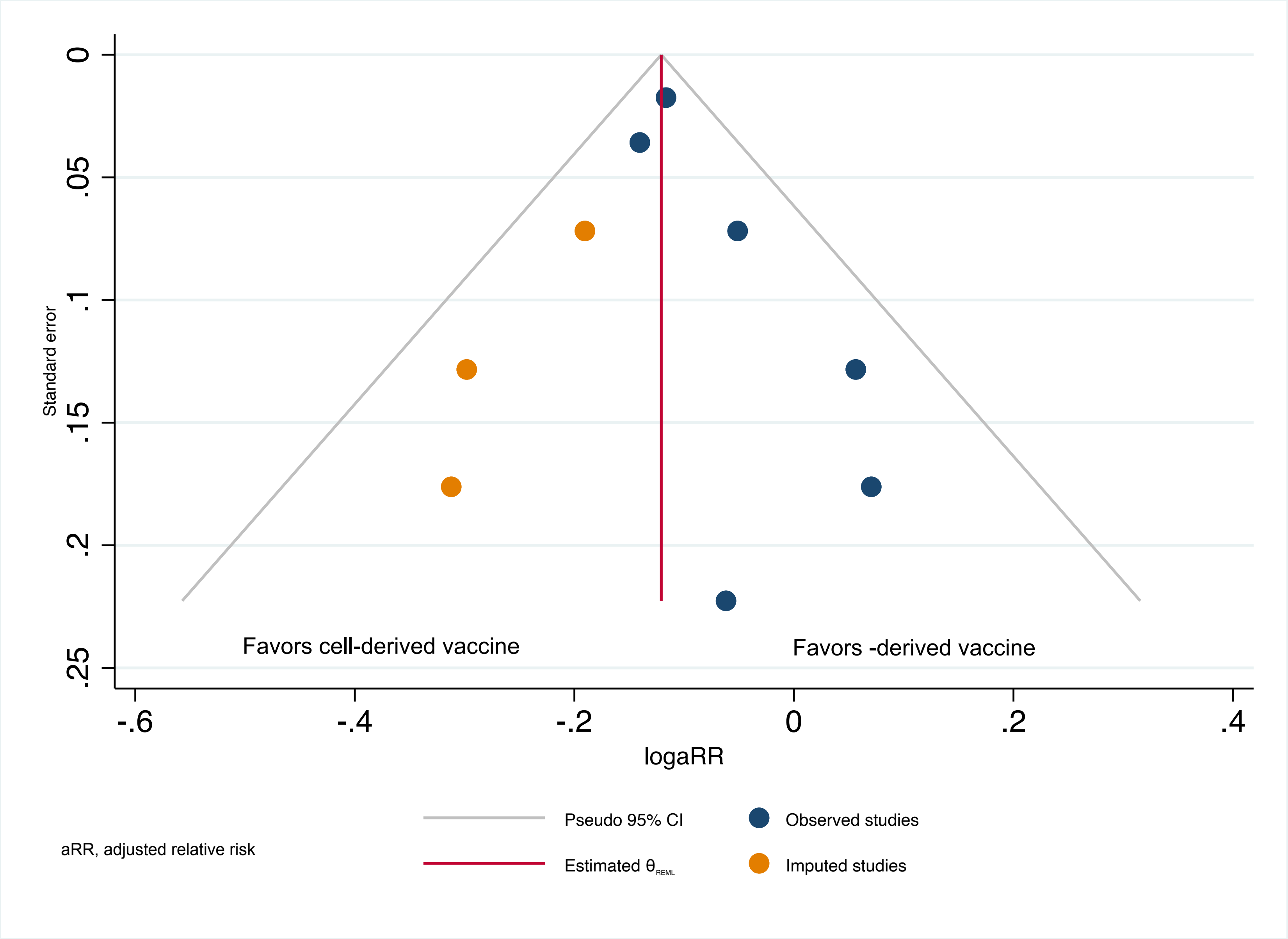

Supplement: Supplementary file 1 [file ijerph-19-00818-s001.zip › Figure_S4_v04.pdf]
