# Supplementary material for: Relative Effectiveness of Cell-Cultured versus Egg-Based Seasonal Influenza Vaccines in Preventing Influenza-Related Outcomes in Subjects 18 Years Old or Older: A Systematic Review and Meta-Analysis
Source: Int J Environ Res Public Health. 2022 Jan 12;19(2):818. doi: 10.3390/ijerph19020818 (PMC8775496; doi:10.3390/ijerph19020818)
Supplement: Supplementary file 1 [file ijerph-19-00818-s001.zip › Table S2 Sensitivity Leave one out analysis v04.pdf]

Table S2 Leave one out analysis, 2017-2018 and 2018-2019.
Random-effects model Method: REML

Season and Omitted study	aRR	[95% conf	interval]	p-value
2017-2018 Influenza season				
Boikos 2020a	0.891	0.865	0.918	0.000
Bruxvoort 2019a	0.892	0.866	0.919	0.000
Divino 2020	0.921	0.858	0.988	0.022
Eick-Cost 2018	0.890	0.863	0.917	0.000
Izurieta 2019b	0.924	0.845	1.010	0.080
Klein 2020a	0.890	0.864	0.917	0.000
Martin 2020c	0.892	0.866	0.919	0.000
exp(theta)	0.892	0.866	0.919	0.000
2018-2019 influenza season				
Boikos 2021c	0.947	0.920	0.975	0.000
Boikos 2021d	0.993	0.952	1.035	0.725
Fu Tseng 2019b	0.971	0.929	1.015	0.192
Izurieta 2020a	0.972	0.917	1.029	0.325
Krishnarajah 2021	0.977	0.927	1.029	0.377
exp(theta)	0.972	0.931	1.015	0.200

aRR, adjusted relative risk
exp(theta) poooled adjusted relative risk
